# Evaluation of Seasonal Heat Stress on Transcriptomic Profiles and Global DNA Methylation of Bovine Oocytes

**DOI:** 10.3389/fgene.2021.699920

**Published:** 2021-10-29

**Authors:** Fabian A Diaz, Emilio J Gutierrez-Castillo, Brittany A Foster, Paige T Hardin, Kenneth R Bondioli, Zongliang Jiang

**Affiliations:** School of Animal Sciences, AgCenter, Louisiana State University, Baton Rouge, LA, United States

**Keywords:** heat stress, bovine, oocyte, gene expression, DNA methylation

## Abstract

Heat stress affects oocyte developmental competence and is a major cause of reduced fertility in heat stressed cattle. Negative effects of heat stress on the oocyte have been observed at morphological, biochemical and developmental levels. However, the mechanisms by which heat stress affects the oocyte at the transcriptional and epigenetic levels remain to be further elucidated. Here we aimed to investigate the effect of heat stress on oocyte quality, transcriptomic profiles and DNA methylation of oocytes collected through the transition from spring to summer under Louisiana conditions. Summer season resulted in a lower number of high quality oocytes obtained compared to the spring season. There was no difference in *in vitro* maturation rates of oocytes collected during spring as compared to summer. RNA sequencing analysis showed that a total of 211 and 92 genes were differentially expressed as a result of heat stress in GV and MII oocytes, respectively. Five common genes (*E2F8, GATAD2B, BHLHE41, FBXO44*, and *RAB39B*) were significantly affected by heat in both GV and MII oocytes. A number of pathways were also influenced by heat stress including glucocorticoid biosynthesis, apoptosis signaling, and HIPPO signaling in GV oocytes, and Oct4 pluripotency, Wnt/beta-catenin signaling, and melatonin degradation I in MII oocytes. In addition, fluorescent immunocytochemistry analysis showed no difference in global levels of DNA methylation and DNA hydroxymethylation at either the GV or MII stage between spring and summer oocytes. The results of this study contribute to a better understanding of the effect of heat stress on the molecular mechanisms altered in bovine oocytes.

## Introduction

Cattle under the effect of heat stress present reduced fertility. Multiple *in vivo* and *in vitro* studies have been performed to understand the effect of heat stress on the oocyte and the implications on development of resultant embryos. Negative effects of heat stress on the oocyte have been observed at morphological, biochemical and developmental levels. It has been shown that the percentage of *Bos taurus* oocytes developing to the 2-, 4-, 8-cell and morula stages were higher during the cool season than in the hot season (Rocha et al., 1998). Oocytes collected in the winter and summer differed in morphology, where approximately 85% of oocytes recovered in winter presented homogenous dark cytoplasm in contrast to non-homogenous dark regions which were found in approximately 65% of summer oocytes (Zeron et al., 2001). The hot season resulted in a higher proportion of oocytes presenting delayed embryonic cleavage and altered gene expression compared to cold season (Gendelman et al., 2010). Oocytes collected during the summer showed altered developmental competence, mitochondrial distribution, and genetic expression of mitochondrial related genes involved in the respiratory chain of metaphase II (MII) oocytes (Gendelman and Roth 2012b). Heat stress can induce alterations in the chromosomes, spindle and cytoplasmic microtubules and microfilaments. The percentage of oocytes affected increased with the length of time of exposure to elevated temperatures (Tseng et al., 2004). Furthermore, it has been evidenced that lactation status influences the effects of heat stress on oocytes and embryos. There was an increased number and higher quality of oocytes obtained from heifers compared to lactating cows during summer. Similarly, a higher blastocyst rate was obtained from oocytes recovered from heifers than lactating cows during summer (Ferreira et al., 2011).

The molecular effects of heat stresses on bovine oocytes have been documented using qPCR-based candidate gene assay (Payton et al., 2011; Gendelman and Roth, 2012a; Gendelman and Roth, 2012b; Gendelman and Roth, 2012c; Ferreira et al., 2016) or hybridization-based microarray approach (Ticianelli et al., 2017; Payton et al., 2018). For example, microarray analysis of heat stressed oocytes has shown altered abundance of multiple transcripts important in mitochondrial function (Payton et al., 2018). Genes involved in oocyte maturation and early embryonic development (*MOS, GDF9* and *POU5F1*) have also been found to present altered expression in MII oocytes when evaluating oocytes collected during the hot season compared to cold season (Gendelman and Roth 2012c). *In vivo* and *in vitro* models of heat stress have shown that heat stressed oocytes present altered gene expression of developmentally important genes (*CX43, CDH1, DNMT1, C-MOS, GDF9, GAPDH, POU5F1* and *HSPA14*) (Gendelman and Roth 2012a). Decreased protein synthesis is observed in heat-stressed oocytes. Importantly, the removal of cumulus cells further reduces protein synthesis during heat-stress (Edwards and Hansen 1996). However, the comprehensive evaluation of seasonal heat stresses on transcriptome profiles of bovine oocytes remains unexplored.

The highly regulated gene expression during oocyte maturation and embryo development requires regulation in cis by transcription factors and regulation in trans by epigenetic mechanisms (Smith and Meissner 2013). DNA cytosine methylation is one of the main epigenetic mechanisms. Drastic DNA methylation reprogramming occurs during gametogenesis and embryogenesis, leading to the conversion of terminally differentiated gametes into a totipotent embryo. The reprogramming process may be influenced by external and internal factors that result in improper DNA methylation in gametes. For example, manipulation of oocytes by superovulation, *in vitro* maturation (IVM), oocyte cryopreservation, etc. influences oocyte competence, largely through the introduction of DNA methylation abnormalities (Zhu et al., 2021). There is growing interest in identifying the methylated regions that are perturbed at susceptible loci leading to aberrant gametes in livestock. The developmental process may also be influenced by environmental stress (e.g., seasonal heat stress) that could result in improper epigenetic changes in gametes.

Collectively, multiple studies have evaluated the effect of heat-stress at multiple levels of oocyte development and maturation, however, the effects of heat-stress on the transcriptomic profiles and DNA methylation of bovine oocytes remain unexplored. In this regard, the objective of this study is to evaluate the effect of seasonal *in vivo* heat stress on the transcriptomic profiles and DNA methylation/hydroxymethylation of bovine oocytes at the Germinal Vesicle (GV) and Metaphase II (MII) stages.

## Materials and Methods

### Experimental Animals and Climatic Data

All procedures in this experiment were approved by the Louisiana State University Agricultural Center Institutional Animal Care and Use Committee. A crossbred group of non-lactating, non-pregnant *Bos taurus* Angus-based beef cows (*n* = 10) with ages ranging between 3 and 6 years were used. The body condition scores of the animals used ranged between 5-7 (6.6 ± 0.69) on a 9-point scale. Experimental samples were obtained from animals housed at the Reproductive Biology Center, LSU Agcenter, Saint Gabriel, LA. Animals had ad libitum access to Bermuda grass (*Cynodon dactylon*) and water, where animals were managed under the same conditions during spring and summer. Climatic information was obtained from the Louisiana State University Ben Hur Farm Climatic station (30.360384,-91.171797) during spring and summer of 2016. Temperatures ranges during spring were between 49 and 77°F and during summer were between 70 and 83°F. The climatic station recorded temperature and relative humidity measurements once hourly resulting in 3,672 climatic observations between April and August. The Reproductive Biology Center is located approximately 10 miles away from the Ben Hur Farm Climatic Station. Temperature Humidity Index (THI) by day was calculated utilizing the equation (NOAA 1976; Mader et al., 2006; Dikmen and Hansen 2009): THI = 0.8 T_db_ + RH x (T_db_-14.4) + 46.4, where; T_db_ is dry bulb temperature (°C) and RH is Relative Humidity in decimal form.

### Ovum Pick-Up

Animals were restrained in a chute, administered a sedative dose (IM, Xylazine, XylamedTM, VetOne^®^, Boise, ID, United States) and an epidural analgesic dose (lidocaine 2%, VetOne^®^, Boise, ID, United States) prior to the OPU procedure. A portable ultrasound equipped with an 8.5 MHz convex transducer (Micromaxx, Sonosite Inc, Bothell, WA) was used for COCs aspiration. Follicles >3 mm were targeted. The aspiration system used consisted of a disposable needle (18G x 3inch, Air Tite Products N183) attached to polyethylene tubing connected to a 50 ml plastic tube (BD falcon, BD Biosciences, Bedford, MA). The negative pressure for aspiration was created through the utilization of an OPU pump (Cook Veterinary Products, Australia) connected to the 50 ml plastic tube and controlled using a foot pedal. Dulbecco’s phosphate buffered saline (DPBS) (Sigma-Aldrich, St. Louis, MO) supplemented with 1% Bovine Calf Serum (vol/vol; Hyclone Laboratories Inc, Logan, UT) and 0.1% Heparin (10,000 USP/ml, Sagent pharmaceuticals, India) was used as collection media for COCs. Following each OPU session per animal, the contents of the 50 ml tube were filtered (EmCon^®^) and rinsed three times with PBS. Contents of the filter were transferred to a gridded square Petri dish (Agtech, Manhattan, KS) and the COCs were identified utilizing a stereoscopic microscope (SMZ-2B, Nikon, Tokyo, Japan). Cumulus-oocyte complexes were transferred to Hepes-TALP, graded and maintained inside an air incubator at 37°C until further processing. Oocyte grading was performed as follows and according to the International Embryo Technologies Society (https://www.iets.org). Grade 1: >5 layers even cumulus cells with even cytoplasm, Grade 2: three to five layers cumulus cells, mostly even distribution, even cytoplasm, Grade 3: < 3 layers dense compact cumulus cells, often uneven investment, abnormally small oocytes with clear, granular and uneven cytoplasm.

### *In vitro* Maturation of Oocytes

A randomly allocated subset of the retrieved COCs (50% of total number collected per animal) were subjected to *in vitro* maturation (IVM). The maturation media consisted of TCM-199 (Sigma-Aldrich, St. Louis, MO) as base medium and was supplemented with 2 mM of L-glutamine (Sigma-Aldrich, St. Louis, MO), 0.2 mM sodium pyruvate (Sigma-Aldrich, St. Louis, MO), Gentamycyn (5 μl/ml, Sigma-Aldrich, St. Louis, MO), FSH (5 ul/ml, Folltropin, Bioniche, Ontario, Canada) and 10% Fetal Bovine Serum (v/v, Sigma-Aldrich, St. Louis, MO). COC rinsing plates (4-well plate dishes) containing 500 µl of IVM medium per well covered with 400 µl of mineral oil and IVM plates (35 × 10 mm Petri dish plates) containing 50 µl drops of IVM medium covered with 3 ml of mineral oil were prepared 12 h in advanced of the IVM procedure. COCs subjected to IVM were rinsed 3-4 times in pre-warmed HEPES-TALP and then rinsed 3-4 times in IVM rinsing plates before being moved to IVM plates. Cumulus-oocyte complexes were incubated in 50 µl drops (10–15 COCs/drop) for 24 h under a 5% CO_2_ atmosphere at 38.5°C. To assess oocyte maturation rate, COC expansion and DAPI staining during fluorescent immunocytochemistry were used to assess nuclear maturation, oocytes containing a metaphase plate and a polar body were considered mature oocytes.

### RNA Sequencing (RNA-Seq) Analysis

Cumulus oocyte complexes (GV stage or MII stage) were subjected to cumulus-cell removal prior to analysis. Briefly, oocytes were incubated with Hepes-TALP supplemented with hyalorunidase (1 mg/ml, Sigma-Aldrich, St. Louis, MO) for 3 min at 37°C and then vortexed at maximum strength for 4 min. Hepes-TALP supplemented with 10% FBS was then added to the tube and vortexed for 20 s. Denuded oocytes were washed with Hepes-TALP. Pools of four oocytes were snap frozen and stored at −80°C until analysis.

The RNA-seq libraries were generated by using the Smart-seq2 v4 kit with minor modification from manufacturer’s instructions. Briefly, pooled oocytes were lysed, and mRNA was captured and amplified with the Smart-seq2 v4 kit (Clontech). RNA-seq libraries were constructed using Nextera XT DNA Library Preparation Kit (Illumina) and multiplexed by Nextera XT Indexes Kit (Illumina). Library quality and quantity were determined by using Qubit 3.0 assay (Life Technologies) and DNA high sensitivity kit with Tapestation 4,200 system (Aglilent), respectively. Pooled indexed libraries were then sequenced on Illumina NextSeq 500 with 100 bp pair-end reads. For RNA-seq data analysis, multiplexed sequencing reads that passed quality control filters were trimmed to remove low-quality reads and adaptors. The quality of reads after filtering was assessed, followed by alignment to the bovine genome UMD3.1.1 by STAR (2.5.3a) with default parameters. Individual mapped reads were quantified to calculate fragments per kilobase of exon per million mapped fragments (FPKM) values. Genes with FPKM >0.1 in at least one sample were kept for downstream analysis, consisting of Pearson correlation, principal component analysis (PCA) and cluster analysis using R (R foundation for Statistical Computing, version 3.6.0.) Differential gene expression analysis was performed by the Partek Flow GSA algorithm with default parameters. The genes were deemed differentially expressed if they provided a *p* value <0.05 and foldchange >2. Expression pattern clusters were generated by the unsupervised hierarchical clustering analysis using R. Gene Ontology (GO) and pathway analysis were performed by using the Ingenuity Pathway Analysis software (IPA, Qiagen).

### Immunostaining of 5-methylcytosine (5mC) and 5-hydroxymethylcytosine (5 hmC) on oocytes.

Germinal vesicle stage and MII stage oocytes utilized for DNA methylation/DNA hydroxymethylation analysis were denuded following the same procedure performed for RNA-seq. For DNA methylation/DNA hydroxymethilation analysis, denuded oocytes were subjected to permeabilization and fixation prior to storage. Briefly, the oocytes were exposed to PBST [pH:7.4; PBS (Gibco, Life Technologies, Carsbad, CA) supplemented with 1 μl/ml of Tween 20 (Sigma-Aldrich, St. Louis, MO), 1 μl/ml Phenol Red (Sigma-Aldrich, St. Louis, MO) and 1% Bovine Serum Albumin (Sigma-Aldrich, St. Louis, MO)] for 1 min at room temperature. Oocytes were then pre-fixed in 0.25% paraformaldehyde (PBS as base solution) for 10 min at 37°C. Following pre-fixation, oocytes were rinsed in PBS for 10 min at 4°C. For final fixation, oocytes were exposed to 88% pre-cooled (−20°C) methanol for 1 min. Oocytes were transferred to 0.6 ml tubes containing 88% pre-cooled methanol and stored at −20°C until the immunostaining procedure.

On day of immunostaining, oocytes stored in 88% methanol at −20°C were allowed to reach room temperature. Oocytes were then incubated in PBST for 1 min and subsequently incubated in 2N HCl for 30 min at 37°C. Following the DNA denaturation step, oocytes were exposed to borate buffer (pH: 8.5; 6.2 mg/ml boric acid in PBS without Ca^+2^ and Mg^+2^) for 1–2 min for HCl neutralization. Oocytes were incubated in PBS with 2% BSA (pH: 7.4) for 1 h at room temperature to prevent non-specific binding of antibodies. Oocytes were incubated with antibodies against 5mC (IgG1 monoclonal mouse antibody 33D3, Thermo-Fisher Scientific, Waltham, MA) at 1:250 dilution and 5 hmC (rabbit serum antibody, Active Motif, Carlsbad, CA) at 1:1,000 dilution for 45 min at 37°C. After primary antibody incubation, oocytes were rinsed 3 times for 15 min each in PBS supplemented with 2% BSA plus 0.05% Tween 20. For secondary antibody incubation, oocytes were incubated with Alexa Fluor 488 (Goat anti-mouse IgG, Life Technologies, Carlsbad, CA) at 1:100 dilution and Alexa Fluor 546 (Donkey anti-rabbit IgG, Life Technologies, Carlsbad, CA) at 1:400 dilution for 45 min at 37°C. Following secondary antibodies incubation, oocytes were rinsed 3 times for 15 min each in PBS with 2% BSA plus 0.05% Tween 20. Oocytes were transferred to microscope slides (3″x1″, 1 mm thick, Thermo Fisher, Waltham, MA) and mounted with Antifade solution containing DAPI (Antifade Gold, Molecular Probes, Life Technologies, Carlsbad, cA) and covered with a coverslip (N^o^1, 18 × 18 mm, VWR, Radnor, PA). Oocytes were equilibrated with antifade solution for 24 h before microscope analysis.

A fluorescent microscope (Eclipse N*i*, Nikon, Tokyo, Japan) equipped with light filters for DAPI (Ex: 377 nm/Em: 447 nm), FITC (Ex: 482 nm/Em: 536 nm) and TRITC (Ex: 543 nm/Em: 593 nm) was used for capturing images for nuclear stage, DNA methylation and DNA hydroxymethylation, respectively. Oocyte images were captured at ×40 magnification utilizing a black and white camera (Zyla sCMOS 5.5 MP, Andor, Belfast, Ireland). Software used for image capturing was Nikon NIS Elements Version 4.40. This software was used for image deconvolution utilizing 2D Landweber deconvolution with 10 iterations.

DAPI staining was used to assess nuclear maturation, oocytes containing a metaphase plate and a polar body were considered mature oocytes; and their fluorescence quantification was accounted for DNA methylation/DNA hydroxymethylation of metaphase II stage oocytes. Fluorescence quantification was performed as described previously by McCloy et al. (2014). Fluorescence intensity of images was quantified utilizing the imaging software ImageJ (National Institute of Health, Bethesda, MD). The software was set to include the calculation of area, mean of grey values and integrated density. The nucleus of the GV stage oocytes or the metaphase plate of the MII stage oocytes was manually outlined to define the fluorescent area. Based on the manually defined fluorescent area, the software calculates the mean of grey values and integrated density. The image background was determined by the average intensity measurement of three random circles in the cytoplasm of the oocytes. Fluorescence quantification is calculated through the following Corrected Fluorescence Intensity (CFI) formula: CFI = Integrated density—(area of nucleus x average background intensity values).

### Experimental Design

A crossbred group of non-lactating non-pregnant *Bos taurus* derived Angus-based beef cows (*n* = 10) were used for sample collection (oocytes). Samples for this study were collected during the spring to summer transition twice monthly (7 days between collections) from april to August. The rationale to collect samples during spring to summer transition was based in literature (Rocha et al., 1998; Gendelman and Roth, 2012a; Gendelman and Roth, 2012b; Gendelman and Roth, 2012c) where cattle present reduced oocyte quantity, quality and developmental competence during the summer. We performed ten OPU sessions (twice monthly, five sessions for DNA methylation analysis and five sessions for RNA-seq) from each of 10 cows. Particularly, samples from the first collection in each month were used for DNA methylation/DNA hydroxymethylation analysis while samples from the second collection were used for RNA-seq. All animals were subjected to dominant follicle removal 5 days before the first sample collection. For each sample collection we obtained at least six oocytes per cow, which resulted in at least three observations (biological sample) per developmental stage per cow per month. GV oocytes collected from each month were graded and recorded. Half of the collected GV oocytes were used for RNA-seq and DNA methylation analysis, and the other half of the GV oocytes were *in vitro* maturated to MII oocytes. Maturation rate was evaluated, and MII oocytes were collected for RNA-seq and DNA methylation analysis as well.

For RNA-seq analysis, oocytes collected from May (spring) and July (summer) were grouped and six pooled oocytes (four GV or MII as a pool, one pool corresponding to one animal) were used for RNA-seq library preparation. For DNA methylation analysis, oocytes (GV or MII oocytes) were grouped as spring (april and May) and summer (June, July and August). In this manner the experimental design resulted in two treatments (spring and summer) x number of oocytes of observations per two developmental stages (GV and MII).

### Statistical Analysis

The Statistical Analysis System (SAS 9.4) software was used for all statistical analyses. Normality of the results was assessed using the Shapiro Wilk test. Temperature, humidity, THI, total oocytes per cow, DNA methylation, DNA hydroxymethylation were analyzed with the Type III test of fixed effects and Tukey media separation using Proc Glimmix, where replication (cow) was considered a random effect. Maturation rates and percent of Grade 1, Grade 2 and Grade 3 oocytes were square root arcsin transformed for statistical analysis. The difference in the proportion of days per month with THI>75 and THI>79 was analyzed by Chi-square. Level of significance was set at *p* < 0.05. For temperature, humidity, THI, total oocytes per cow, maturation rate, DNA methylation/DNA hydroxymethylation statistical analysis, months were grouped as spring (april and May) and summer (June, July and August). Results (Least squares mean [LSM] ± standard error [SE]) were evaluated as spring and summer.

## Results

### Climatic Conditions

Heat stress in cattle is estimated through temperature humidity index (THI). In *Bos taurus* beef cattle, a THI over 75 is an indicator of heat stress (St-Pierre et al., 2003). In this experiment settings, the climatic conditions of temperature, relative humidity, Temperature Humidity Index and number of days with THI over 75 and over 79 are shown in Table 1. All measured climatic conditions including temperature (*p* < 0.0001), relative humidity (*p* < 0.0001), and THI (*p* < 0.0001) are lower during spring compared to summer. There were fewer days with THI over 75 (*p* < 0.0001) and with THI over 79 (*p* < 0.0001) in spring compared to summer (monthly average temperatures, humidity and THI information can be found in Figure S1A and S1B). Additionally, more than half of the days of the summer months had an average humidity index over 75 (Figure S1B).

**TABLE 1:** Climatic conditions during experiment.

Season	Relative				
	Temperature (°F)	Humidity (%)	THI	THI >75	THI >79
Spring	67.34 ± 0.73 ^**a**^	82.84 ± 1.13 ^**a**^	69.17 ± 0.57 ^**a**^	5/61 ^**a**^	0/61 ^**a**^
Summer	77.66 ± 0.33 ^**b**^	87.28 ± 0.63 ^**b**^	77.66 ± 0.21 ^**b**^	85/92 ^**b**^	30/92 ^**b**^

* Different superscripts within a column denote a significant difference between parameters (*p* < 0.05). THI was calculated utilizing temperature values in °C.

### Quantity, Quality and Maturation Rates of GV Oocytes

Overall, a greater number of oocytes were recovered during spring than during summer (*p* = 0.0002) (Table 2). A higher percent of grade 1 oocytes was obtained during spring than during summer (*p* = 0.0183) (Table 2). No difference in percent of grade 2 oocytes was observed between treatments (*p* = 0.9660) (Table 2) but a lower percent of grade 3 oocytes was obtained during spring than during summer (*p* = 0.0016) (Table 2). After *in vitro* maturation of GV oocytes, we found no significant difference in maturation rates between spring and summer (Table 3).

**TABLE 2:** Oocyte grade and total number of oocytes obtained.

Season	Grade %			Total oocytes per cow	Total oocytes
	1	2	3		
Spring	38.25 ± 3.69^a^	21.80 ± 2.44^a^	39.82 ± 4.54^b^	21.88 ± 2.34^a^	422
N	147	93	182		
Summer	27.59 ± 3.09^b^	22.60 ± 2.20^a^	55.87 ± 3.98^a^	14.23 ± 2.17^b^	426
N	102	85	239		

*Different superscripts within a column denote a significant difference between parameters (*p* < 0.05). OPU sessions: five; cows aspirated per season: 10. Grade 1: >5 layers even cumulus cells with even cytoplasm, Grade 2: three to five layers cumulus cells, mostly even distribution, even cytoplasm, Grade 3: <3 layers dense compact cumulus cells, often uneven investment, abnormally small oocytes with clear, granular and uneven cytoplasm.

**TABLE 3:** Maturation rates of oocytes subjected to *in vitro* maturation.

Season	Total	Matured	MII oocytes (%)
Spring	79	65	81.92 ± 4.04 ^**a**^
Summer	123	113	91.11 ± 3.36 ^**a**^

* Different superscripts within a column denote a significant difference between parameters (*p* < 0.05). OPU sessions: five; cows aspirated per season: 10. DAPI staining was used to assess nuclear maturation, oocytes containing a metaphase plate and a polar body were considered matured oocytes.

### Summer Heat Stress Induced Transcriptional Changes in GV and MII Oocytes

To delineate the gene pathways affected by heat stress in oocytes, RNA-seq analysis was conducted on the GV and MII oocytes collected in spring (May) and summer (July) seasons. In total, we analyzed 24 samples and obtained approximately 12.5 million paired-end reads per individual sample (Supplementary Table S1). The raw FASTQ files and normalized gene expression levels are available at Gene Expression Omnibus (GEO) (www.ncbi.nih.gov/geo) under the accession number GSE166413.

**FIGURE 1:**
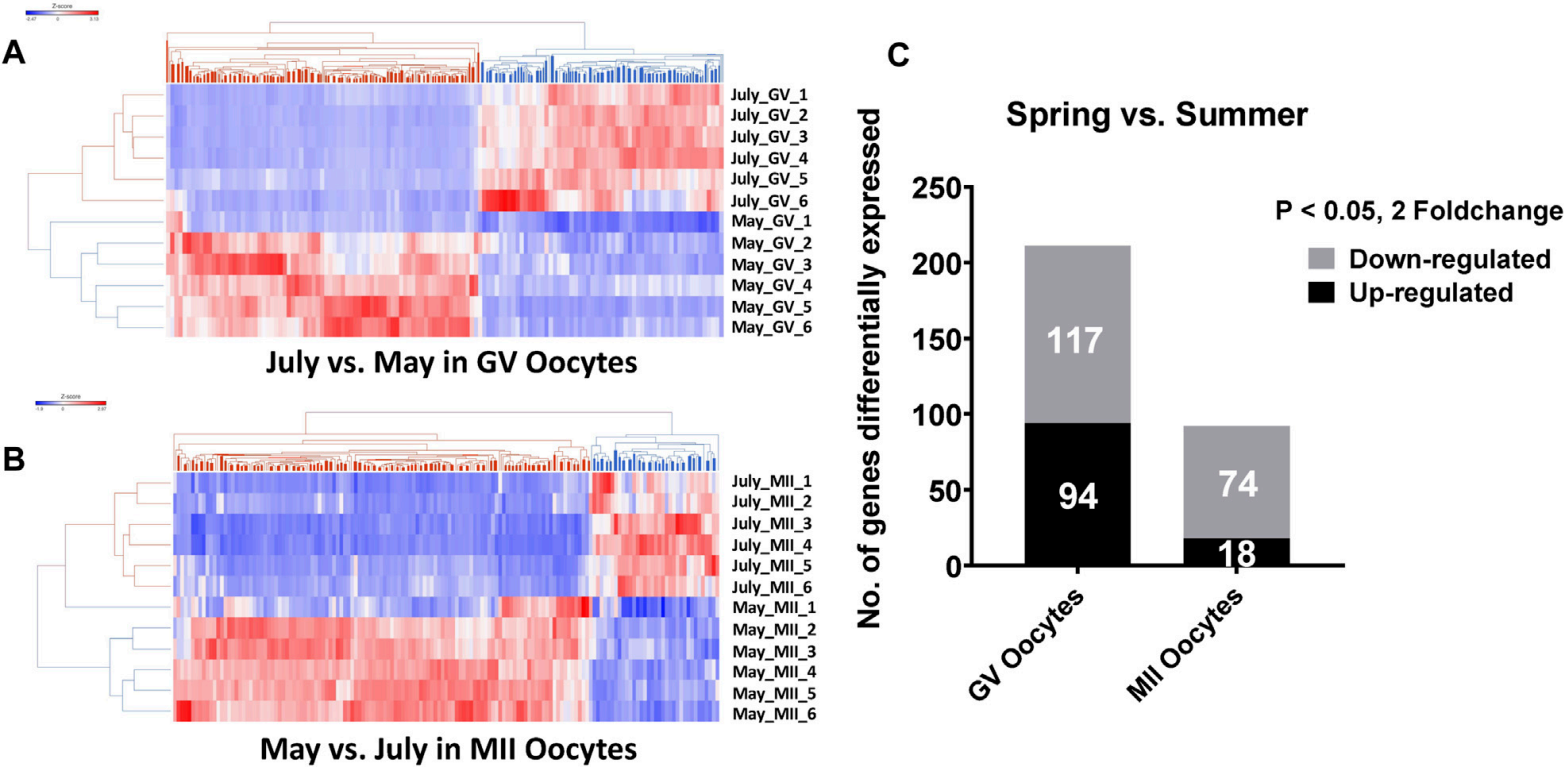
Hierarchical clustering of differentially expressed genes between spring (May) and summer (July) season produced GV oocytes. **(A)** and MII oocytes. **(B)** C. The number of differentially expressed genes between spring (May) and summer (July) season in GV and MII oocytes. RNA-seq analysis was performed in six biological replicates of GV or MII oocytes pool (4 oocytes per group) per season.

**FIGURE 2:**
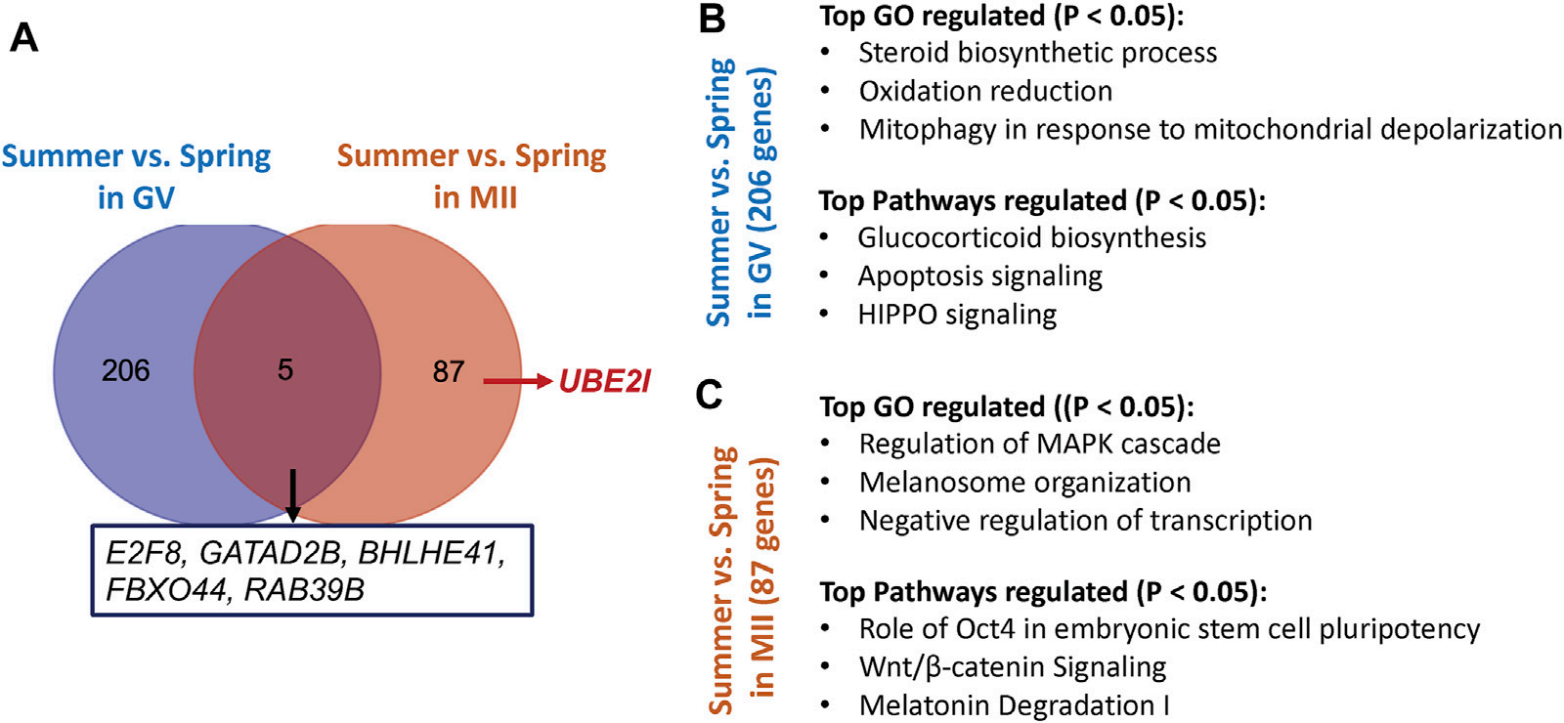
**(A)** The number of genes and their representatives regulated by different season (spring vs summer) in GV or MII oocytes **(B)** The top represented Gene ontology (GO) terms and pathways affected by heat stress in GV oocytes. **(C)** The top represented Gene ontology (GO) terms and pathways specifically affected by heat stress in MII oocytes.

Unsupervised hierarchical clustering of the differentially expressed genes partitioned into two distinct clusters to separate both GV and MII oocytes derived from spring and summer seasons, respectively (Figures 1A,B). A total of 211 genes were differentially expressed as a result of heat stress at the GV stage in summer as compared to spring (94 upregulated and 117 downregulated) (Figure 1C). GO analysis indicated significant over-representation of elements involved in steroid biosynthetic processes, oxidation reduction, and mitophagy in response to mitochondrial depolarization (Figure 2). A number of pathways were influenced by heat stress including glucocorticoid biosynthesis, apoptosis signaling, and HIPPO signaling (Figure 2).

At the MII stage, only 92 genes were significantly differentially expressed when comparing oocytes collected in spring and summer (18 upregulated and 74 downregulated) (Figure 1C). The primary biological processes significantly affected at the MII stage were regulation of the MAPK cascade, melanosome organization, and negative regulation of transcription (Figure 2). In addition, pathways including Oct4 pluripotency signaling, Wnt/beta-catenin signaling, and melatonin degradation I were affected in MII oocytes from spring, compared to summer (Figure 2). Interestingly, we found that *UBE2I*, a gene involved in phosphorylation-dependent sumoylation of transcription factors, was significantly up-regulated in summer compared to spring in MII oocytes (Figure 2A). To note, five common genes were significantly affected by heat in both GV and MII oocytes; *E2F8*, *GATAD2B*, *BHLHE41*, *FBXO44*, and *RAB39B* (Figure 2A).

**FIGURE 3:**
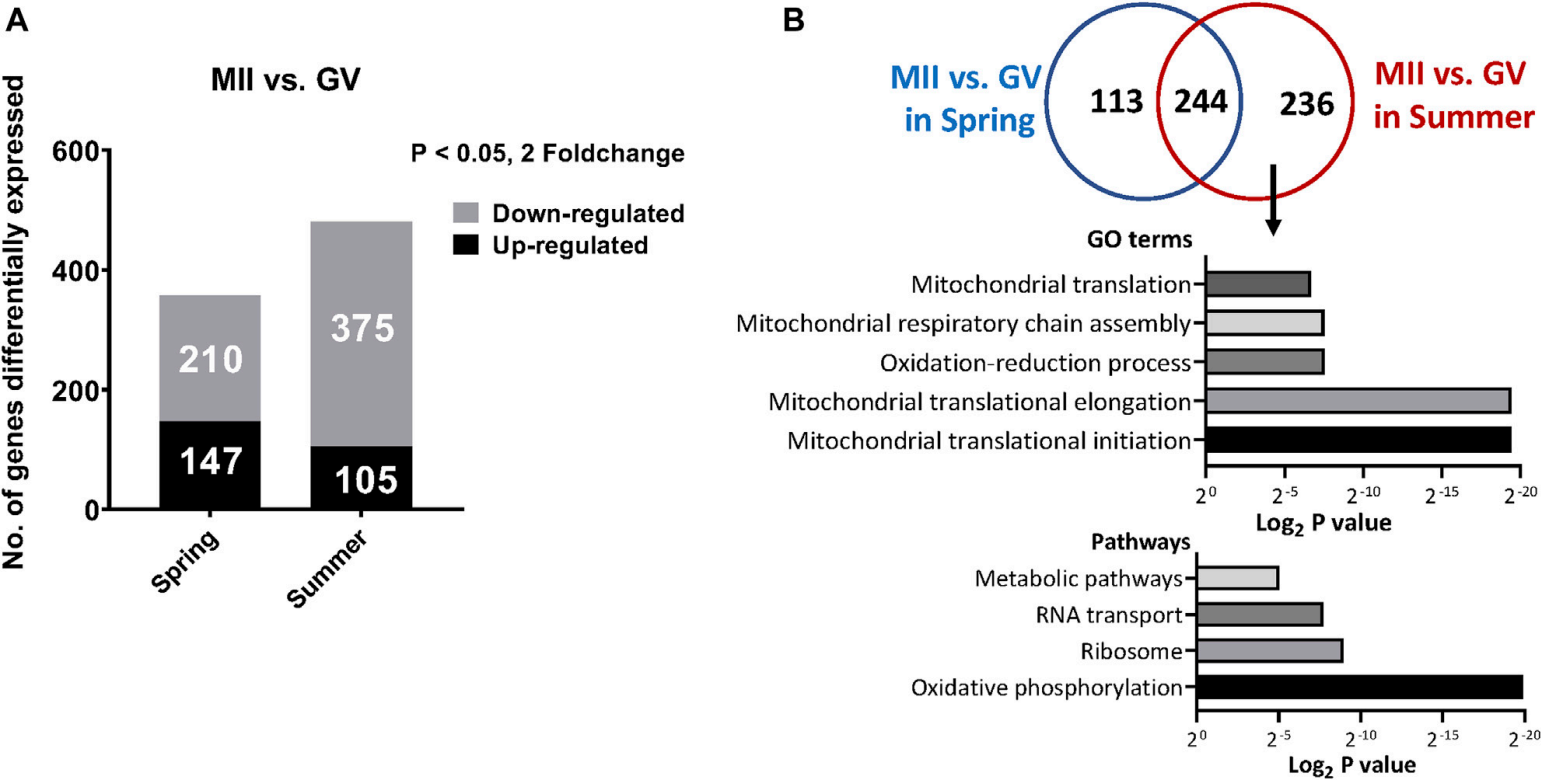
**(A)** The number of differentially expressed genes between GV and MII oocytes collected in spring or summer season **(B)**. Commonly and specifically differentially expressed genes between MII vs GV oocytes in spring or summer seasons and the top represented GO terms and pathways specifically affected by heat stress from Summer during *in vitro* maturation.

We also determined the genes differentially expressed between MII and GV oocytes from spring or summer seasons. A total of 357 and 480 genes were significantly differentially expressed between MII and GV spring and summer, respectively (147 upregulated and 210 downregulated in spring; 105 upregulated and 375 downregulated in summer) (Figure 3A). Among these genes, 236 differentially expressed genes were specifically affected by heat stress from summer during *in vitro* maturation. GO analysis of these genes showed they were involved in mitochondrial translation initiation/elongation, mitochondrial respiratory chain assembly and oxidation reduction processes (Figure 3B). Pathway analysis revealed oxidative phosphorylation, ribosome, RNA transport, and metabolic pathways were significantly regulated (Figure 3B).

### The Effect of Heat Stress on DNA Methylation of Oocytes

**FIGURE 4:**
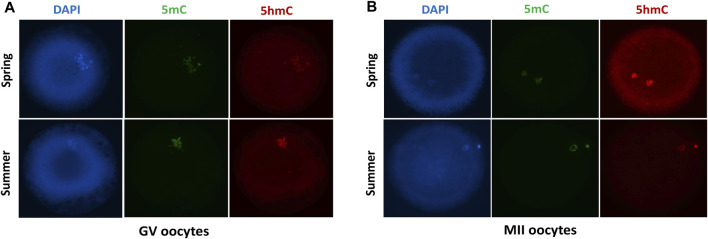
Immunofluorescent staining with DAPI, DNA methylation (5mC) and DNA hydroxymethylation (5 hmC) of GV **(A)** and MII **(B)** oocytes collected during spring and summer.

We determined the global DNA methylation and DNA hydroxymethylation at GV (Figure 4A) and MII oocytes (Figure 4B) recovered during spring and summer by immunostaining analysis. We found no difference in DNA methylation and DNA hydroxymethylation between spring and summer recovered GV oocytes (Table 4). We performed additional immunoassays of MII oocytes following *in vitro* maturation. Similarly, no significant difference was found in DNA methylation and DNA hydroxymethylation between spring and summer (Table 5).

**TABLE 4:** Relative fluorescence levels of DNA methylation and DNA hydroxymethylation of GV stage oocytes.

Season	N	DNA methylation	DNA hydroxymethylation
Spring	67	417,218.90 ± 71,793.86 ^**a**^	444,931.10 ± 67,920.78 ^**a**^
Summer	149	313,819.88 ± 55,528.01 ^**a**^	35,254.68 ± 56,425.96 ^**a**^

* Different superscripts within a column denote a significant difference between parameters (*p* < 0.05). OPU sessions: 5; cows aspirated per season: 10.

**TABLE 5:** Relative fluorescence levels of DNA methylation and Hydroxymethylation of metaphase II oocytes.

Season	N	DNA methylation	DNA hydroxymethylation
Spring	60	87,122.36 ± 14,449.47 ^**a**^	102,933.83 ± 15,517.70 ^**a**^
Summer	111	89,807.26 ± 11,303.72 ^**a**^	137,622.45 ± 11,826.86 ^**a**^

* Different superscripts within a column denote a significant difference between parameters (*p* < 0.05). OPU sessions: five; cows aspirated per season: 10.

## Discussion

In this study, a significantly higher number of oocytes per cow were obtained in spring compared to summer. In addition, a higher percentage of high quality (Grade 1) oocytes was also obtained during spring, while a higher percent of low quality (Grade 3) oocytes was obtained during summer. These results are consistent with findings of previous studies. Rocha et al. (1998) collected oocytes through OPU and found that the total number of oocytes collected and the proportion of oocytes classified as normal were higher during the cold season compared to the hot season. Similarly, when collecting oocytes from abattoir derived ovaries from Holstein cows, the total number of oocytes and the incidence of homogeneous dark cytoplasm are higher during the winter compared to summer months (Zeron et al., 2001; Gendelman et al., 2010). Torres-Júnior et al. (2008) found that heat stress increased the number of denuded/degenerated COCs recovered from *Bos indicus* cattle. Nevertheless, they did not find differences in the total number of oocytes obtained either in the Grade 1 or Grade 2 oocytes between heat stressed and control groups. Therefore, heat stress negatively affects the quality and the number of oocytes available for collection through OPU or abattoir derived ovaries.

There was no difference in vitro maturation rates of oocytes during the spring and summer in our study. Conflicting evidence has been reported on the effects on heat stress on oocyte maturation. Multiple studies have reported that *in vitro* heat stress has resulted in reduced maturation rates (Lenz et al., 1983; Payton et al., 2004; Maya-Soriano et al., 2013; Pavani et al., 2015). Similarly, *in vivo* heat stress has resulted in reduced proportion of MII stage oocytes (Pavani et al., 2015). However, there are studies where no difference in maturation rates was found between heat stressed oocytes and control groups (Edwards et al., 2005; Roth and Hansen, 2005; Hooper et al., 2015). One possible explanation of the discrepancy between studies might be the variation of breeds used for oocyte collection. For example, differences in the tolerance to heat stress have been seen not only between *Bos indicus* and *Bos taurus* cattle, but also among breeds of the same species (St-Pierre et al., 2003; Liao et al., 2018). It is noteworthy that most of the data available have been obtained from abattoir derived oocytes and the effects of heat stress have been mainly studied using *in vitro* models for heat stress. Whereas, an *in vivo* heat stress model was used in our study where we retrieved oocytes through OPU from cows exposed to natural environmental conditions of high temperature humidity index. Although *in vitro* models of heat stress might be valuable in providing information when collection of oocytes and embryos from live donors might not be possible, the most reliable and accurate information on the effects of heat stress on reproduction and animal fertility are obtained through studies utilizing live animals with naturally occurring heat stress conditions. In addition, there is a lack of consensus regarding the temperatures and time of exposure used for the induction of *in vitro* heat stress between studies. Higher temperatures and longer exposure result in decreased developmental competence and maturation rates.

In this study, the effect of seasonal heat stress on gene expression profiles of bovine oocytes was comprehensively evaluated using RNA-seq. We identified a total of 211 and 92 differentially expressed genes regulated as a result of heat stress in GV and MII oocytes, respectively. In GV oocytes, the differentially expressed genes are involved in steroid biosynthetic processes, oxidation reduction, and mitophagy in response to mitochondrial depolarization. The pathways influenced by heat stress are related to glucocorticoid biosynthesis, apoptosis signaling, and HIPPO signaling. In MII oocytes, the differentially expressed genes are involved in regulation of the MAPK cascade, melanosome organization, and negative regulation of transcription. The pathways influenced by heat stress are related to Oct4 pluripotency signaling, Wnt/beta-catenin signaling, and melatonin degradation. Interestingly, we found that *UBE2I* was significantly up-regulated in summer compared to spring in MII oocytes. *UBE2I* is a gene involved in phosphorylation-dependent sumoylation of transcription factors. Loss of oocyte *UBE2I* has been found to cause defect in oocyte- and granulosa cell expressed genes in mouse (Rodriguez et al., 2019). This phenomenon resulted in female infertility with defects in stability of primordial follicle pool, ovarian folliculogenesis, ovulation and meiosis (Rodriguez et al., 2019). Additionally, we found that five genes were commonly regulated by heat stress in both GV and MII stage oocytes, including *E2F8, GATAD2B, BHLHE41, FBXO44,* and *RAB39B*.

Few studies have documented the genes affected by heat stress in bovine oocytes. For example, Ferreira et al. (2016) observed decreased expression of *NRF1, POLG, POLG2, PPARG-C1A, TFAM, BAX, ITM2B, FGF16,* and *GDF9* in GV oocytes aspirated from Holstein cows during the summer compared to those aspirated in the winter. Genes such as *MOS, GDF9, POU5F1,* and *GAPDH* had higher expression levels in MII oocytes after *in vitro* heat stress (Gendelman and Roth, 2012c). Similarly, a higher expression of *C-MOS, GDF9, POU5F1,* and *GAPDH* was observed in MII oocytes retrieved during the cold season compared to the hot season (Gendelman and Roth, 2012a). A reduced expression of genes involved in mitochondrial energy production including *ND2, SDHD, CYTB, COXII,* and *ATP5B* was reported in MII oocytes obtained during the summer compared to winter (Gendelman and Roth, 2012b). Ticianelli et al. (2017) evaluated the gene expression profile, through microarray analysis, of in vitro heat shocked Holstein and Nelore OPU derived oocytes. Nine genes were found differentially expressed; three up-regulated: *ACOX1, TTC8, UPF3A*, and six down-regulated: *RPLP1, MT1E, FAU, SF3B4, COR O 1C, C11ORF16*. Breed × Temperature interaction analysis revealed up-regulation of other genes including *CCT4, DICER1,* and *OSMR*. Payton et al. (2018) also evaluated the effects of in vitro heat stressed abattoir derived oocytes on transcriptome profile using microarray analysis. Particularly they reported a model highlighting the possible impacts of heat-stress on mitochondrial related processes. Further analysis of three transcripts related to oxidative phosphorylation confirmed the findings revealed by microarray showing a reduction of polyadenylated mRNA of heat-stressed oocytes. These signaling have also been regulated in bovine oocytes under our heat stress conditions. We also revealed additional pathways that affected by the seasonal heat stress on the bovine oocytes, which providing additional insights of how heat stress affects the oocyte competence.

Our results indicate no difference in global DNA methylation nor DNA hydroxymethylation levels of GV or MII stage oocytes aspirated in the spring and summer. Although heat stress does not alter global DNA methylation and DNA hydroxymethylation in GV and MII oocytes as detected by immunostaining assays, the methylation status of specific gene loci in oocytes induced by heat stress need to be further determined by sequencing-based approaches at genome-wide resolution.

## Conclusion

Environmental stress reduces oocyte developmental competence and is a major cause of reduced fertility in cattle. In this study, we assessed the effects of seasonal heat stress on bovine oocytes quality, transcriptomic profiles, and global DNA methylation with an *in vivo* heat stress model. We found that summer season resulted in a lower total number and lower high quality GV oocytes obtained compared to the spring season. There is no difference on the *in vitro* maturation rate of GV oocytes collected during spring and summer. RNA sequencing analysis revealed a substantial number of genes and pathways are regulated by the seasonal heat stress both in the GV and MII oocytes, which provide new insights of molecular mechanisms leading to aberrant oocytes. Although neither the global levels of DNA methylation nor DNA hydroxymethylation of GV or MII oocytes obtained between the spring and summer differs, further genome-wide bisulfite sequencing approach is warranted to reinforce the seasonal heat stress effects on DNA modifications at gene specific loci.

## Data Availability

The datasets presented in this study can be found in online repositories. The names of the repository/repositories and accession number(s) can be found below: https://www.ncbi.nlm.nih.gov/geo/, GSE166413.
