# Supplementary figures and images for: Evaluation of Seasonal Heat Stress on Transcriptomic Profiles and Global DNA Methylation of Bovine Oocytes
Source: Front Genet. 2021 Oct 29;12:699920. doi: 10.3389/fgene.2021.699920 (PMC8585773; doi:10.3389/fgene.2021.699920)

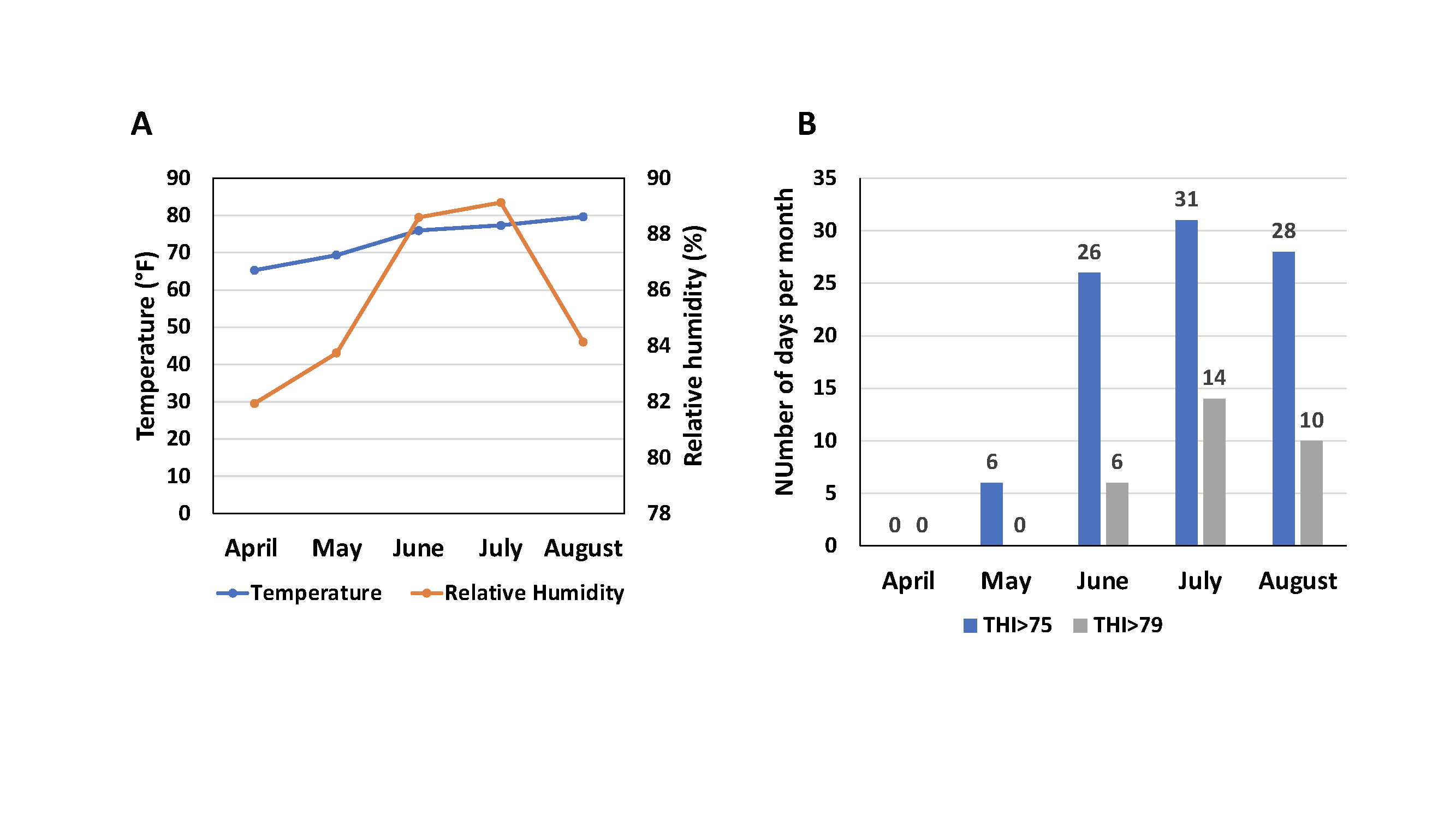

Supplement: Supplementary file 1 [file Image1.TIFF]
